# Do Men Have No Need for “Feminist” Artificial Intelligence? Agentic and Gendered Voice Assistants in the Light of Basic Psychological Needs

**DOI:** 10.3389/fpsyg.2022.855091

**Published:** 2022-06-14

**Authors:** Laura Moradbakhti, Simon Schreibelmayr, Martina Mara

**Affiliations:** Robopsychology Lab, Linz Institute of Technology, Johannes Kepler University, Linz, Austria

**Keywords:** agency, autonomy, Artificial Intelligence, gender stereotypes, voice assistants, competence, relatedness, technology acceptance

## Abstract

Artificial Intelligence (AI) is supposed to perform tasks autonomously, make competent decisions, and interact socially with people. From a psychological perspective, AI can thus be expected to impact users’ three Basic Psychological Needs (BPNs), namely (i) autonomy, (ii) competence, and (iii) relatedness to others. While research highlights the fulfillment of these needs as central to human motivation and well-being, their role in the acceptance of AI applications has hitherto received little consideration. Addressing this research gap, our study examined the influence of BPN Satisfaction on Intention to Use (ITU) an AI assistant for personal banking. In a 2×2 factorial online experiment, 282 participants (154 males, 126 females, two non-binary participants) watched a video of an AI finance coach with a female or male synthetic voice that exhibited either high or low agency (i.e., capacity for self-control). In combination, these factors resulted either in AI assistants conforming to traditional gender stereotypes (e.g., low-agency female) or in non-conforming conditions (e.g., high-agency female). Although the experimental manipulations had no significant influence on participants’ relatedness and competence satisfaction, a strong effect on autonomy satisfaction was found. As further analyses revealed, this effect was attributable only to male participants, who felt their autonomy need significantly more satisfied by the low-agency female assistant, consistent with stereotypical images of women, than by the high-agency female assistant. A significant indirect effects model showed that the greater autonomy satisfaction that men, unlike women, experienced from the low-agency female assistant led to higher ITU. The findings are discussed in terms of their practical relevance and the risk of reproducing traditional gender stereotypes through technology design.

## Introduction

People in the industrialized world increasingly rely on Artificial Intelligence (AI) to obtain information, get personalized recommendations, or make decisions in their day-to-day life. In professional contexts, for example, doctors now use intelligent image analysis tools for diagnoses (Davenport and Kalakota, 2019; Kaplan et al., 2021) and HR managers at companies let algorithms preselect who should be invited to a job interview (Liem et al., 2018; Houser, 2019). In personal life, data-driven AI systems recommend movies according to the user’s preferences (Lawrence, 2015; Floegel, 2020), monitor sleeping patterns (Alqassim et al., 2012; Lee and Finkelstein, 2015; Kolla et al., 2016), allow you to chat as you would do with a friend and provide emotional support (e.g., Replika, replika.ai), manage your home *via* smart home technologies (Robles and Kim, 2010; Wilson et al., 2017; Gram-Hanssen and Darby, 2018; Marikyan et al., 2019), and support your every-day banking tasks (Letheren and Dootson, 2017; Li et al., 2020). Through the use of machine intelligence, data are analyzed faster than ever before, decision processes are accelerated, monotonous tasks can be handed over to the computer, and in cases of chatbots and speech assistants, sociable connections are possible without a real human dialog partner being involved.

As new technologies emerge, we quickly adapt to them and integrate them into our personal and professional life. Yet, from a psychological point of view, we need to question how they impact human experience, motivation, and well-being. According to the Basic Psychological Needs Theory (BPNT; Deci and Ryan, 2000; Ryan and Deci, 2000), which has received a lot of attention in fields other than technology use, motivation to engage in a task and subsequent well-being can be achieved through (i) personal autonomy, (ii) the feeling of being competent, and (iii) relatedness to other people.

Looking at current developments and new applications offered in AI, which often involve competent and autonomous decision-making or building social connections, it can be argued that AI systems may target the three spheres addressed by BPNT. Nevertheless, empirical research to date has hardly investigated the association between behavioral intentions to use AI-based applications and the perceived fulfillment of the three Basic Psychological Needs (BPNs), nor how variations in system designs or user-specific factors play a role here.

The present study is therefore dedicated to the question of need fulfillment in the interaction with an AI-based smartphone assistant, as a function of (a) more vs. less agency of the assistant, (b) female vs. male perceived design features of the assistant, and (c) user gender. In doing so, we aim not only to inspire greater consideration of BPN in the future design of AI systems, but also to take up the current discourse around gender-stereotypical design of AI-based voice assistants (West et al., 2019).

## Basic Psychological Needs Theory

As part of their Self-Determination Theory of human motivation (SDT), Deci and Ryan (1985, 2008) developed six mini-theories, one of which is the BPNT (Deci and Ryan, 2000; Ryan and Deci, 2000). According to the authors, if a task or situation leads to satisfaction of the three BPN—autonomy, competence, and relatedness—it creates human autonomous motivation to engage in the task, greater well-being, and overall satisfaction. If all three needs are fulfilled, we are motivated to perform an action, and we lack autonomous motivation, if one or more of these needs remain unfulfilled. Within the theoretical framework,

- *Autonomy* relates to our desire to have control over a situation and our actions,
- *Competence* refers to our innate desire to experience mastery over a task, and
- *Relatedness* is our need to care for others and be cared for in return.

Over the recent years, BPN Fulfillment was measured most commonly with the Basic Psychological Need Satisfaction and Frustration Scale (BPNSFS; Chen et al., 2015). In comparison with previous scales, such as the Balanced Measure of Psychological Needs (BMPNs, Sheldon and Hilpert, 2012) or the Basic Psychological Need Satisfaction Scale (BPNS; Ilardi, 1993), the BPNSFS measures Need Satisfaction and Need Frustration as two separate constructs. Need Frustration was added to the construct, as needs can not only be satisfied but also actively blocked (Chen et al., 2015). While Need Satisfaction relates to the prediction of well-being, Need Frustration contributes to ill-being (Chen et al., 2015). Conceptually, this means that the lack of Need Satisfaction does not necessarily equal Need Frustration (Tindall and Curtis, 2019). According to Longo et al. (2018), Need Satisfaction and Need Frustration should not be measured as part of a continuum but as two different constructs. Assessing Need Frustration and Satisfaction as two separate constructs yielded better construct reliability. Relatedness Satisfaction is the feeling of having a connection to others and Relatedness Frustration refers to loneliness; Competence Satisfaction is closely connected to the feeling of effectiveness and capability, while Competence Frustration relates to failure; lastly, Autonomy Satisfaction is linked to volition and Autonomy Frustration to the feeling of being controlled (Chen et al., 2015).

Typically, the BPNT is referred to in the context of work (Deci and Ryan, 2014; Williams et al., 2014; Ilies et al., 2017), education (Tian et al., 2014; Wang et al., 2019), or physical activity (Balaguer et al., 2012; Gunnell et al., 2013; Li et al., 2013), where the BPN have been described as influential drivers of motivation and well-being. For example, participants who found the three BPN to be fulfilled at work, while controlling for job status and pay, also reported higher self-esteem, greater overall satisfaction with their job, and even less psychosomatic symptoms (Ilardi, 1993). In contrast, burnout was found to be negatively predicted by the BPN (Li et al., 2013). These findings indicate the importance of psychological need satisfaction for mental and physical well-being. In a consumer research context, empirical evidence indicates that a sense of autonomy and self-determination can positively affect consumers (André et al., 2018). For the technology sector, a user experience study concluded that the fulfillment of the needs for relatedness and competence (in addition to stimulation and popularity as further drivers) led to positive affect and a positive perception of interactive gadgets such as mobile phones, mp3 players, and navigation devices (Hassenzahl et al., 2010).

As our interactions with AI technologies intensify, we propose that they may also have an impact on our BPNs, and that the extent to which our needs are satisfied plays a critical role in our motivation to use and engage with such new technologies. We consider each of the three BPN as highly relevant to the context of AI. With regard to the Need for Relatedness, research shows that robots can help to reduce loneliness (Ghafurian et al., 2021) and lead to attachment to AI systems in times of social isolation (Xie and Pentina, 2022). Contrarily, it has been reported that use of technologies can to lead to an increase in social isolation (Muhammad et al., 2019). It is thus important to assess which characteristics of AI systems may foster or hinder users’ Relatedness Satisfaction. Relevant to the Need for Autonomy, policy makers such as the High-Level Expert Group on Artificial Intelligence (HLEG-AI) established by the European Commission name the support of human autonomy in decision-making a key requirement for public acceptance of AI (HLEG-AI, 2018). This illustrates the importance of assessing user’s Autonomy Satisfaction—user’s need to make independent decisions and have control over daily tasks—while interacting with AI. Ignoring AI’s impact on user’s feelings of Autonomy Satisfaction can have negative effects (Chen et al., 2015; André et al., 2018). Therefore, AI should be designed in a way that, even though it is highly autonomous and independent, the user’s autonomy is not undermined. Lastly, AI is designed to be efficient, relieve people of working on monotonous tasks, and “making our life easier.” Nonetheless, it may be counterproductive to develop systems that neglect a user’s own feeling of mastery and competence. As a recent study has shown, users’ Competence Satisfaction is influenced by understanding the AI assistant’s capabilities and the effectiveness of the conversation, suggesting again that design factors of AI play a crucial role in the need satisfaction of users (Yang and Aurisicchio, 2021).

Interestingly, empirical research that investigates the role of BPN on AI acceptance is scarce to date. Recently, one of the first studies to draw a link between BPN Fulfillment and AI has investigated chatbot-assisted decision-making (De Vreede et al., 2021). The results revealed that a stronger experience of autonomy, competence and relatedness was indeed associated with higher user satisfaction, which subsequently led to greater engagement with the chatbot. In line with these findings is a proposed research model by Nguyen and Sidorova (2018) that also highlights the importance of BPN consideration to achieve system satisfaction with websites and chatbots. A third study assessed the influence of the three BPN together as a variable named “self-determined interaction” on customer experience with a chatbot (Jiménez-Barreto et al., 2021). As their results revealed, self-determined interaction had a positive influence on customer experience, user satisfaction, and attitudes toward the chatbot.

Overall, based on evidence from other domains as well as initial empirical findings in the field of human–computer interaction, we expect that the BPN play a role in user responses to AI assistants and their intention to interact with such technology. The extent to which individuals feel their needs for autonomy, competence, and relatedness fulfilled when using an AI assistant could account for individual differences in the acceptance of such systems. While the aforementioned empirical studies focused on interactions with text-based chatbots, there is still a research gap in the domain of—increasingly popular—AI-based voice assistants. Moreover, no empirical work to date has looked at BPN Fulfillment in Human–AI interaction as a function of gendered features or agency levels of a technology. Therefore, our experiment is the first to manipulate these two design factors of an AI-based voice assistant and relate them to BPN Fulfillment and technology acceptance. Both factors that our study investigates, agency and gender, are relevant to the default settings of popular voice assistants on the market (often a combination of female voice and low agency) and are shaping human interaction with them. In the following, we review relevant literature on agency and gender in technology design.

## Review of the Literature: Agency and Gender

### AI Assistants and Their Agency

Agency refers to the (perceived) capacity for self-control of an AI assistant (e.g., Gray et al., 2007). Autonomous or semi-autonomous AI systems often have a high level of agency, as they make decisions and take actions independently, on the basis of data analyses. In contrast, AI systems with low levels of agency require the constant input of human commands or guidance. As studies suggest, agency can influence the perception of a non-human agent (Appel et al., 2020; Brink and Wellman, 2020; Zafari and Koeszegi, 2020). In Human–Robot Interaction research, agency has been linked to increased anthropomorphism (the tendency to infer human-like traits to non-human entities; Nowak and Biocca, 2003; Epley et al., 2008; Crowell et al., 2019). Just as individual differences occur in the perception of anthropomorphism (Epley et al., 2008), agency perception and subsequently agency preferences for non-human agents vary due to individual differences (Stafford et al., 2014; Brink and Wellman, 2020). Finding the right balance of activity and passivity between the AI assistant and the user is an important issue in the design of Human-AI interactions (Zafari and Koeszegi, 2020; Pizzi et al., 2021).

On the one hand, users have been found to prefer a proactive style when interacting with a chatbot (Thies et al., 2017; Chaves and Gerosa, 2020). Proactivity can be defined by the level of initiative a chatbot shows with the user, for example, by creating a more natural conversation (Morrissey and Kirakowski, 2013) or by adding new topics and asking follow-up questions (Chaves and Gerosa, 2020). On the other hand, users crave some sense of control over an AI system’s actions and may feel controlled (Chaves and Gerosa, 2020) or threatened if it behaves too autonomously (Maedche et al., 2019; Stein et al., 2019). For example, in a study, participants were asked to view videos of human-robot collaborations and put themselves in the shoes of the person in the video (Zafari and Koeszegi, 2020). When robots exerted high agency, meaning the human had low levels of control in the tasks, they were perceived more negatively in comparison with robots exerting low agency.

These results suggest that high-agency levels of AI assistants could have both a negative and positive impact on the fulfillment of BPN. While there is some empirical work on the perception of non-human agents with gender features as a function of more or less agency (see section “Gender-Specific Differences in Agency Level Preferences”), there is still a literature gap regarding the effects of machine agency on BPN Fulfillment, especially in the new field of AI voice assistants for day-to-day use. An AI assistant with high levels of agency could be experienced as a threat or hindrance for the users to fulfill their own Needs for Competence and Autonomy. However, if users perceive the bot less as a competitor but more as a supportive resource for themselves, a highly agentic AI assistant could also serve as a catalyst for their BPN Fulfillment. As mentioned above, individual differences in the perception of AI assistants may be one way to explain this ambivalence.

### Gendered Designs of AI Assistants

We argue that the transmission of societal concepts such as gender onto machines is one aspect that could account for individual differences in the perception of AI assistants and subsequently need satisfaction and behavioral intentions. According to the Computers Are Social Actors (CASAs) paradigm, humans apply social categories to computers and use cues, such as voice gender to do so (Nass and Moon, 2000). Therefore, people do not only interact with computers in a similar way they would interact with other humans; they also apply social rules and existing gender stereotypes to non-human entities (Reeves and Nass, 1996; Nass and Moon, 2000). Interestingly, it has become a standard to set the default voice of a speech assistant as female (Cambre and Kulkarni, 2019). One question which arises with this standard choice is whether it truly relies on the user’s preference, or whether it just mirrors the traditional societal stereotype of an assistant to be female (Weisman and Janardhanan, 2020).

#### Application of Gender Stereotypes to AI Systems

Gender stereotypes are popular overgeneralized beliefs regarding supposedly typical traits of each gender (Eagly and Wood, 2012). Traditionally, traits that are related to *agency* (e.g., ambitious, assertive, competent, dominant, independent) are stereotypically associated with men (Bakan, 1966; Abele et al., 2008; Hentschel et al., 2019). Traits that are related to *communion* (e.g., caring, emotional, friendly, gentle, understanding) are stereotypically associated with women (Bakan, 1966; Abele et al., 2008; Hentschel et al., 2019). Negative consequences of gender stereotypes, for example, on professional and educational opportunities for women, or backlash effects on nonconforming individuals, have been demonstrated in many studies (e.g., Carr and Steele, 2010; Appel et al., 2011; von Hippel et al., 2011).

Research demonstrates that gender stereotyping applies not only to humans but also to robots (Eyssel and Hegel, 2012) and other non-human agents (Forlizzi et al., 2007). Even disembodied chatbots (Brahnam and De Angeli, 2012; Chaves and Gerosa, 2020) and computer voices (Nass et al., 1997) are perceived and categorized according to gender stereotypes that are traditionally attributed to men and women. Furthermore, it has been found that people tend to apply gender stereotypes if the field of application is traditionally associated with one gender and, in particular, if the chatbot does not act in accordance with its expected gender role (McDonnell and Baxter, 2019). Female-featured chatbots are more likely than male featured chatbots to be attributed with negative stereotypes (e.g., low competence) and to be the recipients of both implicit and explicit sexual language (Brahnam and De Angeli, 2012). Recent reports discuss anecdotal evidence about consumers who use sexually abusive language when addressing “female” speech assistants such as Siri and Alexa (Curry and Rieser, 2018; West et al., 2019). A study by Weisman and Janardhanan (2020) indicated that the use of female-sounding voice assistants—unlike male-sounding voice assistants—had both short- and long-term implications for the treatment of female subordinates in workplace settings: After using a female-featured voice assistant, help from female subordinates was expected to be given more quickly, they were penalized more harshly if they made mistakes, and were spoken to more impersonally than male subordinates. Further research is needed to better understand differential effects of AI design factors that influence the application of gender stereotypes to voice assistants, particularly due to the lack of prior research drawing a connection between the BPNT and gender stereotypes in technology design,

#### Nonconformity With Gender Stereotypes in AI Systems

As outlined above, emerging evidence supports the relevance of research on impacts of gendered technology designs. Strategies that help to avoid the reproduction of societal stereotypes and gender biases in and through AI have received increased attention in recent years (cf. West et al., 2019). To promote gender equality and fairness in AI, suggestions include that datasets from which AI systems learn must be examined for inherent gender bias, AI literacy should be fostered especially among females, and teams developing AI systems should become more diverse and inclusive (cf. Jobin et al., 2019). Given that the stereotypical pairing of female gender markers (female names, voices) and passive, servant roles of AI-based speech assistants has been widely problematized (cf. West et al., 2019), counter-stereotypical designs of AI assistants could complement these strategies, since confrontation with stereotype-incongruent information has the potential to weaken a person’s access to stereotypic associations (e.g., Dasgupta and Asgari, 2004; Finnegan et al., 2015). Such non-conformity with stereotypes may be expressed in technology design, for example, by a female-featured speech assistant that is high in agentic traits. To the best of our knowledge, no previous study has yet examined user responses to stereotype-conforming vs. non-conforming design characteristics of gendered AI assistants in a controlled and randomized study, nor related them to individual differences in BPN Fulfillment based on user gender.

Supporting female agency and dismantling disadvantages that result from prevailing gender stereotypes have always been integral to the feminist movement (e.g., Farrell, 1995; Leaper and Arias, 2011). Feminist identity has been found to be associated with gender role atypicality and criticism of stereotypical depictions of the genders (van Breen et al., 2017). Individuals who identify with feminism also showed a greater likeability of describing themselves with agentic attributes (Saunders and Kashubeck-West, 2006) and to include agentic themes when narrating about their lives (Boytos et al., 2020). Drawing on these findings, in the context of the current study, we take the liberty of referring to a high-agency female AI assistant as the “feminist” condition in our experiment.

#### Voice Gender Preferences in Natural vs. Synthetic Speech

With regard to human preferences of voice gender, it should be noted that, starting in infancy, there seems to be a preference for the female voice (Standley and Madsen, 1990). Stereotypically, females are labeled as warm, tender and sensitive, whereas males are more likely to be described as dominant, assertive and forceful (Brems and Johnson, 1990; Guo et al., 2020). These stereotypical perceptions also align with the perception of female speech (Wiley and Eskilson, 1985; Ko et al., 2006), which is generally attributed with greater likeability (Krahé et al., 2021) and kindness (Ko et al., 2006) than male speech. Such socially favorable perceptions of female-sounding voices, in addition to early habituation to the mother’s voice, have been suggested as major drivers behind frequently found preferences for female speech. Alongside societal changes such as the growing role of fathers in early parenting or more balanced gender distributions in leadership positions, it could be assumed that disparities in evaluations of female and male voices might decrease over time. Today, however, customer preferences for female voices are still frequently put forward as an argument for the selection of female communicators in social interaction domains. This is reflected, for example, in a disproportionate share of female workers in call centers (Fernandez and Sosa, 2005), but also in the female-sounding default setting of many contemporary voice assistants.

For synthetic speech, previous research does not show consistent findings. In a study, both female and male participants preferred male over female synthetic voices and found the former to be more persuasive than the latter (Mullennix et al., 2003). The female synthetic voice was rated “less powerful,” “squeakier,” “softer,” and “faster” than the male voice. In contrast, participants rated the male voice as more positive. Nonetheless, other research in the context of human voices and chatbots found that male and female users differ in their preferences for dominance and persuasiveness. In a recent study (Guo et al., 2020), females had no preference for the gender of a chatbot that was trying to motivate them to pay overdue debt. Male participants, in contrast, were more likely to be persuaded by female chatbots. Furthermore, the study revealed that stereotypically feminine attributes, such as being gentle and warm, positively influenced male customers, while stereotypically male attributes (being forceful and assertive) had negative influence on both male and female customers. These results suggest favorable responses to behavior of chatbots that conform with traditional gender stereotypes. In addition to this, the results are in support of findings from previously outlined studies, proposing an innate human preference for female voices (Standley and Madsen, 1990; Ko et al., 2006; Krahé et al., 2021). Since the findings up to date are ambiguous, the results of the current study will shed further light on voice gender preferences for AI assistants.

#### Preferences for Same-Gender Interaction With AI Assistants

Another line of research suggests same-gender preferences for synthesized speech (Lee et al., 2000, 2007) and thus supports the Similarity Attraction Theory by Byrne (1971), which posits that people are generally more attracted to others who are similar, rather than dissimilar, to themselves. These findings could be particularly relevant for the Satisfaction of the Need for Relatedness, as a study has shown that participants felt more psychological closeness when they interacted with a same-gender robot (Eyssel et al., 2012). In a study examining preferences of synthesized speech with children, the same effect was shown: children were asked to sit in front of a computer and listen to passages that would introduce them to different topics which were spoken by a synthetic voice. The topics were divided into stereotypically male and female topics, such as “make up” and “princesses” or “dinosaurs” and “knights.” Conforming to stereotypical portrayals of gender-specific interests, children preferred either the voice gender that matched the content of the topic or the voice gender that matched their own gender (Lee et al., 2007). These findings are also in line with the presumed desirability of same-gender interaction in HCI as proposed by Lee et al. (2000). However, since this evidence is not in line with other findings as outlined above (Mullennix et al., 2003; Guo et al., 2020; Krahé et al., 2021), additional research needs to be conducted to get a clearer picture of causal mechanisms behind differential responses to gendered technology. To date, it has not been investigated whether attraction to similarity—in this case, to a nonhuman agent whose gender a user identifies with—is associated with higher BPN Fulfillment. The current study will provide new insights on whether users have a higher Intention to Use and Relatedness Satisfaction for voice assistants that match their own gender.

#### Gender-Specific Differences in Agency Level Preferences

With regard to individual differences of users’ preferences for agency levels, we argue that one important factor of influence is in fact existing gender stereotypes. For natural human speech, Carli (1990) demonstrated that male listeners were influenced more by tentatively speaking females, while females were influenced more by female speakers who spoke assertively. For synthetic speech, a study from Taiwan (Chang et al., 2018) revealed that participants exhibited a general preference for an assistive device having an extroverted female synthetic voice, with some individual differences depending on participants’ demographics or personality traits. On the contrary, a study by Nass et al. (1997) found that a female sounding computer voice was perceived as less competent in comparison with a male computer voice, and in addition to this, a female dominant voice was perceived more negatively than a male dominant voice. These findings provide evidence that gender stereotypes are indeed influential for the perception of synthetic speech. In line with gender-stereotypical behavior, female-featured AI assistants may be expected to act less dominant in comparison with male featured ones (Brems and Johnson, 1990; Guo et al., 2020). In reference to widely used systems such as Apple’s Siri and Amazon’s Alexa, there has been increased criticism in recent years over the reproduction of traditional gender stereotypes through the default combination of female gender markers, for example, female names and voices, with low levels of agency, conforming to stereotypical gender roles (Broverman et al., 1972) and outdated portrayals of women as passive servants (West et al., 2019).

Taken together, the majority of evidence either suggests a same-gender preference or a preference for female speech. With regard to agency levels, the small amount of existing research proposes that gender-stereotypical behavior—i.e., a combination of low agency or high communality with feminine features—is preferred by male users. Given the conflicting evidence (Nass et al., 1997; Chang et al., 2018), more research needs to be conducted to get a better understanding of individual differences with regard to gender and agency level preferences of speech assistants. Even though the outlined research does address gender preferences and differences with regard to voice gender and agency levels, none of the studies have focused on BPN Satisfaction in relation to these factors.

## The Current Study

The current study examines the relationship between the satisfaction of BPN, considered here as a function of gender and agency of an AI assistant, and technology acceptance, operationalized as participants’ behavioral Intention to Use (ITU) the AI assistant. As indicated by previous findings, individuals differ in their preferences for and dislikes of computer systems based on gendered design cues (Chang et al., 2018), speech (Carli, 1990), and interaction style (Thies et al., 2017; Maedche et al., 2019; Pizzi et al., 2021) of the system. One aspect that could account for these individual differences might be the gender of the user (Guo et al., 2020).

Even though only a few studies have investigated the role BPN play in technology acceptance, recent findings suggest them to be linked to user satisfaction and engagement with a given technology (De Vreede et al., 2021). As the BPNT (Deci and Ryan, 2000; Ryan and Deci, 2000) and empirical work on BPN indicate, need satisfaction is an important foundation of behavioral motivation (e.g., to engage in a certain task or use a certain product). Complementing existing work, the present study is the first to investigate user’s BPN satisfaction as a function of two distinct design factors of an AI assistant, namely agency and gender. Until now, the factors of agency and gender were not assessed in relation to users’ BPN Fulfillment. Furthermore, the current study draws a link between the design factors, BPN Satisfaction, and participants’ behavioral intentions to use and engage with AI assistants. In the present study, participants were shown a video of an AI-based finance coach for everyday banking support. The AI assistant’s voice (female vs. male) and its agency level (high vs. low) were manipulated across four conditions.

The primary research question for the current study was whether the agency level and “gender” of the AI finance coach would impact the BPN satisfaction of the user and consequently the ITU the finance coach. In line with previous findings that suggest users do not want chatbots to behave too autonomously (Maedche et al., 2019; Stein et al., 2019), we hypothesize a similar effect for the agency level of the finance coach in the current study. We assume that the high-agency finance coaches lead to lower Autonomy Need Satisfaction (higher Autonomy Need Frustration) and thus lower ITU ratings in comparison with the low-agency conditions of the finance coach. Since previous research on preferences with regard to agency in combination with the gender of a voice is contradictory (Carli, 1990; Nass et al., 1997; Chang et al., 2018), we want to further examine the role of the finance coach gender with regard to participants’ Autonomy Need Satisfaction and ITU.

*H1*: High-agency finance coaches lead to lower Autonomy Need Satisfaction (higher Autonomy Need Frustration) and thus lower ITU ratings in comparison with the low-agency conditions of the finance coach.

In line with the Similarity Attraction Theory and some evidence for same-gender preferences of an AI assistant (Lee et al., 2000, 2007; Eyssel et al., 2012), we further test whether a finance coach which matches the user gender (independent from agency levels) would lead to higher Relatedness Satisfaction (lower Relatedness Frustration) and subsequently higher ITU scores.

*H2*: A finance coach which matches the user gender leads to higher Relatedness Satisfaction (lower Relatedness Frustration) and subsequently higher ITU scores.

With regard to the Need for Competence, we assume an influence of Competence Satisfaction (Competence Frustration) on the ITU scores. However, the results of the agency conditions on Competence Satisfaction could go in both directions. Since recent research has pointed at Competence Satisfaction being linked to greater engagement with a chatbot (De Vreede et al., 2021), we assume a positive impact of Competence Satisfaction on ITU. However, the high-agency conditions could either lead to lower Competence Satisfaction (higher Competence Frustration) as participants see their own competence lower in comparison with the high-agency finance coaches’ competence; on the other hand, the high-agency finance coaches could empower the participants and thus satisfy their Competence Need.

*H3*: We assume a positive association between Competence Satisfaction and ITU.

*H4*: Competence Satisfaction (Competence Frustration) differs between high-agency conditions and low-agency conditions.

Lastly, in line with previous research on voice perception (Brems and Johnson, 1990; Nass et al., 1997; Guo et al., 2020) we assume that the male finance coaches will be perceived as more dominant and competent in comparison with the female finance coaches and the high-agency finance coaches will be perceived as more dominant and competent as compared to the low-agency finance coaches, as previous findings have shown that highly-autonomous chatbots can be perceived as controlling and threatening (Maedche et al., 2019; Stein et al., 2019; Chaves and Gerosa, 2020).

*H5*: Male-sounding finance coaches will be perceived as more dominant and competent in comparison with the female finance coaches.

*H6*: High-agency finance coaches will be perceived as more dominant and competent as compared to the low-agency finance coaches.

## Materials and Methods

A 2 × 2 between-subjects design is used to investigate the effects of an AI assistant’s perceived gender (female-sounding/male-sounding voice) and level of agency (high/low) on participants’ satisfaction or frustration of their BNP, related user perceptions of the AI assistant, and intentions to use it.

### Participants

A G*Power (version 3.1.9.6) analysis (*f* = 0.25, power = 0.90) was run to define the sample size. According to the analysis, the minimum sample size needed for the study is 270 participants. In total, 314 participants from either Germany or Austria took part in the experiment, 14 of whom were excluded because their indicated age was below 16 years—and they would therefore have been unable to relate to the topic of financial savings. A further 15 participants were excluded because they did not complete the study or indicated that they had not answered all questions sincerely (see procedure). In addition to this, three further participants were excluded for not having heard the video content properly. The final sample consisted of 282 participants (154 males, 126 females, two non-binary participants, M_age_ = 47.34 years, SD_age_ = 17.63, Range_age_ = 16–94). With regard to the highest level of education, 42% of participants indicated to have completed an apprenticeship or vocational training, 23% completed A-level equivalent education, 10% a bachelor’s degree, 17% a master’s degree or equivalent qualifications, 2% a doctorate degree or higher degrees, and below 2% did either complete a different education or no education. Participants were recruited through the online panel provider *Respondi*. We requested approximately equal gender distribution and a wide age range. In accordance with our requests, *Respondi* emailed participants from their respondent pool with a link to take part in our online study. Participants received €0.55 as an incentive for their participation.

### Procedure

Totally, 263 participants completed the online survey without pausing (M_time_ = 13 min 56 s), and 19 resumed after one break. Prior to taking part in the study, all participants were instructed to either use headphones or keep their computer/laptop audio on high volume for the duration of the study. The entire study was conducted in German. First, participants read an introduction, confirmed their consent, and filled out demographic information. Following these initial steps, a short instruction appeared, which was followed by a finance coach video. Participants were randomly assigned to one of four video conditions (see section stimuli). After each video, the dominance perception and competence perception questionnaires followed in random order before the BPNSFS and ITU scales were displayed. Participants were subsequently asked four control questions to make sure that they had clearly understood the content of the video, that their German language skills allowed them to understand the content of the survey, that they had watched the whole video (even though it was technically impossible to skip the video), and that they had answered all questions honestly. Finally, participants were shown a debrief page before being redirected to Respondi’s platform to process their financial compensation.

### Stimuli

**Figure 1:**
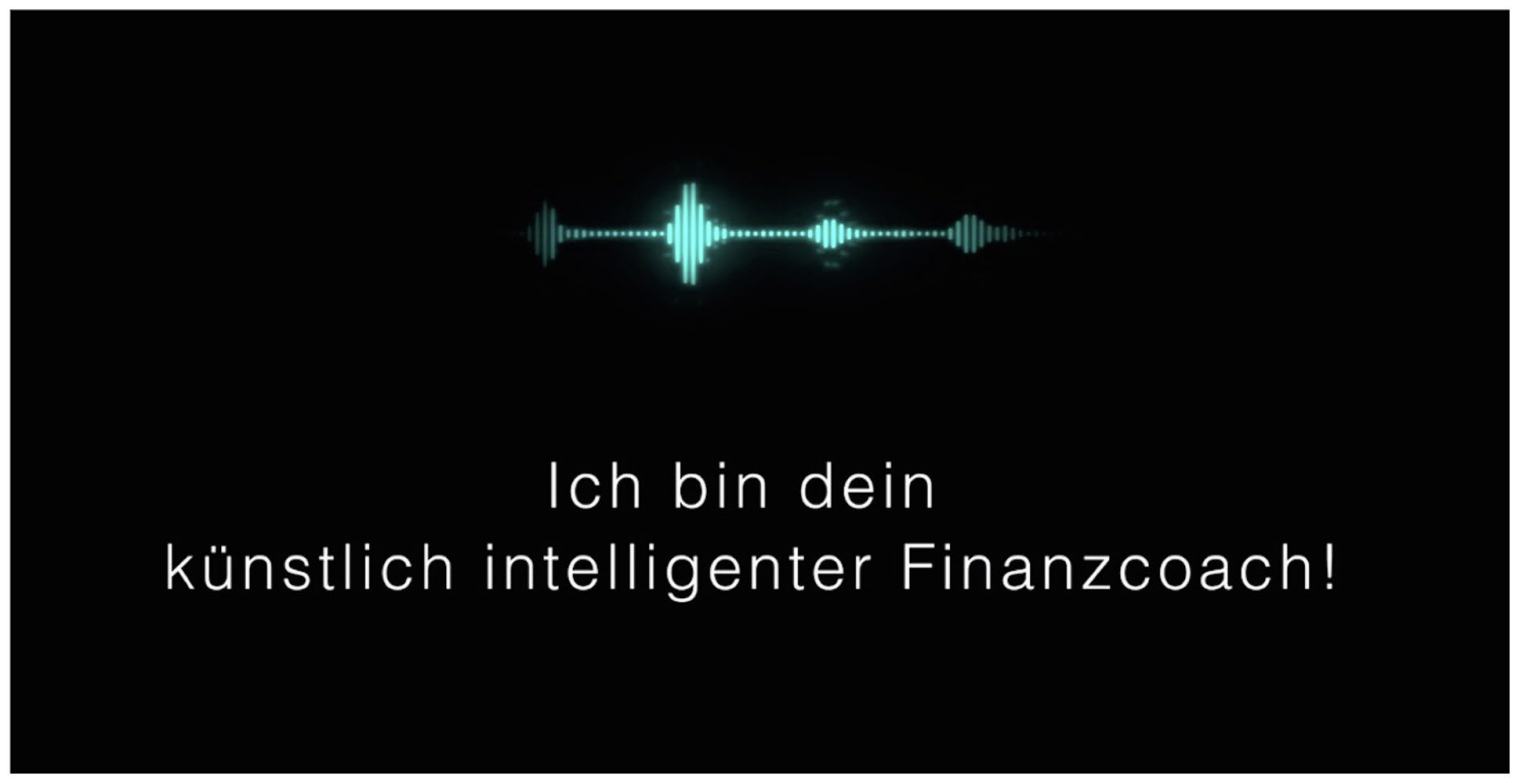
Screenshot from a finance coach video including a text passage and a visualized sound wave form.

In our stimulus videos, an AI assistant for personal banking (“finance coach”) was simulated to introduce its services (e.g., financial analyses) to the participant. We used four videos that differed either in the gender of the finance coaches’ voice (female-sounding/male-sounding) or in the spoken content (indicating low/high agency). The four videos were created using the software Adobe After Effects. In order to emphasize the difference between the low and high agency conditions, particular words and phrases of the spoken text were visualized and highlighted in the video (e.g., “at your request” for low agency and “without your intervening” for high agency; for more detail, see section Appendix A). In addition to this, a sound wave form was shown to visualize the voice of the finance coach in the video (see Figure 1). Total length was 1 min and 53 s in the high-agency condition and 1 min and 52 s in the low-agency condition.

### Independent Variables

#### AI Assistant Agency

The finance coach’s agency level was either high or low. To achieve this manipulation, its services and approach to the customer differed depending on the agency condition. In the low-agency condition the finance coach stated, for example: “If you connect me with your account information, I can help you to manage deposits, planned savings and other tasks,” while the same service would be introduced by “I will autonomously connect with your account information. Without effort on your part, I will take care of deposits, planned savings and other tasks” in the high-agency condition (translated from the original German text). A total of eight phrases were adapted and differed between the conditions to achieve the required variations in agency-level. The full German text and the English translation of the text for both conditions are found in Appendix A.

#### AI Assistant Gender

We manipulated the synthetic voice of the finance coach to indicate male or female gender. The female version was based on a text-to-speech sample from the online platform ttsmp3.com (which offers text-to-speech downloads powered by AWS Polly) where we selected the voice “Vicki”. In order to keep the voice characteristics such as speaking rate and intonation as constant as possible, the recording of the female voice was modified by lowering the pitch to create a male voice with similar properties. Using the software package Ableton Live Suite (10.1.6) and the implementation ICRAM Trax Vocal Transformer, we converted the original text-to-speech output of the female voice into a male voice. With the help of the default filter settings “woman to man,” a frequency range of 146.67–270.00 Hz, a mean of 220.0 Hz, and a tuning (A) of 444.0 Hz were used. Additionally, the pitch was lowered by 8.4%. A total of four male versions were created that differed in the percentage by which the pitch was lowered and which were subsequently evaluated by six independent evaluators. The version that sounded the most male and was at the same time closest to the emotional expression and characteristics of the female voice was selected for this study. All recordings were cleaned with a custom noise removal filter using the software package Audacity and adjusted to the same volume by normalizing the amplitude (see other software descriptions above). All speech samples were in German.

### Dependent Variables

#### Basic Psychological Need Satisfaction and Frustration

The participants’ Need Fulfillment was measured with the BPNSFS. The scale used in this study was built based on a short scale created by Chen et al. (2015). The translation derived from the German adaptation by Heissel et al. (2019). Overall, 12 items were used (four per psychological need). Six items related to Need Satisfaction (two per psychological need) and six items were related to Need Frustration (two per psychological need). The items were also adapted to fit our context. All questions began with “If I used the finance coach for my personal banking operations…” To give an example, a Competence Need Satisfaction item was: “…I would feel competent to perform my banking operations,” and an Autonomy Need Frustration item was: “…I would feel pressured to do things that I would not have chosen myself.” (translated from the original German items). A full list of the adapted items used in this study is found in Appendix B. The participants rated their Need Satisfaction and Frustration for each item on a five-point Likert scale ranging from 1 (not at all) to 5 (very much). Based on previous literature (Chen et al., 2015; Longo et al., 2018), the frustration and satisfaction should be interpreted as separate constructs. In our case, the Frustration indices did not reach Cronbach’s alpha level > 0.66. The Autonomy Frustration index reached a Cronbach’s alpha level of 0.33, the Competence Frustration index reached a Cronbach’s alpha level of 0.66, and the Relatedness Frustration index reached a Cronbach’s alpha level of 0.42. Therefore, we will only continue with analyses of the Satisfaction indices. The Autonomy Satisfaction index reached a Cronbach’s alpha level of 0.74, the Competence Satisfaction index reached a Cronbach’s alpha level of 0.85, and the Relatedness Satisfaction index reached a Cronbach’s alpha level of 0.72. We can also report a good model fit based on Confirmatory Factor Analysis (CFA): RMSEA = 0.078; SRMR = 0.022; CFI = 0.988; TLI = 0.970.

#### Dominance Perception

We asked the participants to rate their perception of the finance coach’s dominance in a five-point semantic differential format. The items included were adapted from Mehrabian & Russel’s “dominance” component of their semantic differential scale (Mehrabian and Russell, 1974; see also Bradley and Lang, 1994 and Mara and Appel, 2015). We used the following four pairs of items: influential—influenced; controlling—controlled; dominant—submissive; autonomous—guided. The dominance scale showed good reliability with a Cronbach’s *α* of 0.72. Based on CFA fit indices, the model is not a good fit: RMSEA = 0.350; SRMR = 0.108; CFI = 0.742; TLI = 0.225. Therefore, we will not include the dominance perception scale in further analyses.

#### Competence Perception

To measure competence perception of the finance coach (e.g., Bergmann et al., 2012), we asked participants to rate the finance coach on a five-point Likert scale—from 1 (not at all) to 5 (very)—using five traits (competent, experienced, intelligent, efficient, and capable). The competence perception scale showed high reliability with a Cronbach’s *α* of = 0.94. We can also report a good model fit based on CFA: RMSEA = 0.056; SRMR = 0.012; CFI = 0.997; TLI = 0.993.

#### Intention to Use

Here, we used two of the ITU items from the Technology Acceptance Model (TAM3; Venkatesh and Bala, 2008) and slightly adapted them to the context of our experiment: “I can imagine using the finance-coach in the future.” and “I would like to be informed about products that are similar to the finance-coach.” Reliability of the items was high, with a Cronbach’s α of 0.87.

## Results

For analysis of our data, we used the statistics software SPSS (version 27) and the PROCESS macro for SPSS (Hayes, 2018). Levels of significance were set at the standard value of *p* < 0.05. As most of the data failed to meet normal distribution criteria, nonparametric tests such as Spearman’s rank-order correlations and Kruskal–Wallis group comparisons were performed. For a large amount of the analyses, we had to exclude the two non-binary participants since our analyses were based on gender comparisons with the voice gender (female/male) of the finance coach. We did not exclude individual outliers, since bootstrapping and nonparametric tests are robust analyses.

### Zero-Order Correlations Between the Dependent Variables

Initial Spearman’s rank-order correlations indicate that participants’ ITU was positively associated with the fulfillment of all three BPNs, whereas the greatest correlation was found for the Need for Competence (*r_s_* = 0.700, *n* = 280, *p* < 0.01), followed by the Need for Autonomy (*r_s_* = 0.654, *n* = 280, *p* < 0.01), and continued by the Need for Relatedness (*r_s_* = 0.587, *n* = 280, *p* < 0.01). Consistent with our assumptions, this suggests that, overall, BPN satisfaction represents a relevant factor for user acceptance of AI assistants. Significant interrelations were moreover observed among the three BPN as well as with perceived competence in such a sense that the more competent the AI assistant was evaluated, the more likely respondents were also to regard it as satisfying their needs and to use such an AI assistant themselves (see Table 1).

**Table 1:** Spearman’s correlations between the dependent variables.

Measure	*M*	*SD*	1	2	3	4	5	6	7
1. Intention to Use	2.20	1.17							
2. Autonomy Need Satisfaction	2.61	1.00	0.654^**^						
3. Competence Need Satisfaction	2.53	1.08	0.700^**^	0.708^**^					
4. Relatedness Need Satisfaction	2.28	0.97	0.587^**^	0.673^**^	0.657^**^				
5. Competence Perception	3.08	1.03	0.599^**^	0.577^**^	0.595^**^	0.557^**^			
6. User Gender	1.55	0.50	0.118^*^	0.067	0.106	0.003	−0.058		
7. Artificial Intelligence (AI) Assistant Gender	0.50	0.50	0.022	−0.017	−0.033	−0.037	−0.084	0.115	
8. AI Assistant Agency	0.51	0.50	−0.027	−0.155^*^	−0.016	−0.018	−0.025	0.070	−0.014

* *p* < 0.05;

** *p* < 0.01 (two-tailed).

User Gender was coded as 1 for women and 2 for men. AI Assistant Gender was coded as 0 for men and 1 for women. AI Assistant Agency was coded as 0 for low agency and 1 for high agency. Two non-binary participants were excluded from analyses for the comparison between User Gender and AI Assistant Gender (*N* = 280).

The significant correlation between Competence Satisfaction and Competence Perception (*r_s_* = 0.595, *n* = 280, p < 0.01) indicates that participants’ own Need for Competence was not negatively but positively affected by the competence perception of the finance coach.

Overall, there was no significant correlation between the gender of the finance coach (AI Assistant Gender) and the other variables. However, the agency level of the finance coach (AI Assistant Agency) negatively correlated with autonomy satisfaction (*r_s_* = −0.155, *n* = 280, *p* < 0.05). This result suggests that higher agency is linked to less autonomy satisfaction which is in line with our hypothesis. See Table 1 for an overview of all zero-order correlations.

### Overall Evaluations and Main Effects

We predicted that the four finance coach types would lead to differences in how much respondents perceived their three BPNs to be fulfilled, how dominant and competent they perceived the system to be, and how much they would be willing to use it.

A Kruskal–Wallis H test showed that there was a statistically significant difference in Autonomy Satisfaction between the four finance coach conditions *χ*^2^(3) = 8.98, *p* = 0.03, with a mean Autonomy Satisfaction score of 2.62. Pairwise comparisons indicated a difference in the Autonomy Satisfaction scores between the female low-agency and the female high-agency finance coach conditions (*p* = 0.042, Bonferroni-adjusted). The female low-agency finance coach resulted in a significantly higher mean Autonomy Satisfaction score (*M* = 2.81, *SD* = 0.98) than the female high-agency finance coach (*M* = 2.39, *SD* = 0.99). There was no evidence of a significant difference in the Autonomy Satisfaction scores between the other conditions.

Kruskal–Wallis H tests indicated no statistically significant overall differences in the Competence Satisfaction ratings [*χ*^2^(3) = 1.116, *p* = 0.773], the Relatedness Satisfaction ratings (χ2(3) = 0.910, *p* = 0.823), the Competence Perception [*χ*^2^(3) = 2.902, *p* = 0.407], and the reported ITU [*χ*^2^(3) = 0.415, *p* = 0.931] between the four finance coach conditions. Related descriptive statistics is found in Appendix C. We refrained from analyzing group differences in Dominance Perception due to poor fit indices of the corresponding scale (see section Dominance Perception).

### Evaluations Within Female and Male User Groups

Next, we looked at potentially different evaluations of the four finance coach variants within the male and female participant subgroups (Unfortunately, only 2 non-binary participants took part in the current study. Since this number is too low to form a third subgroup for statistical analyses, we had to exlcude the 2 non-binary participants. We further discuss this aspect in the limitations and outlook section of the current study).

For male participants, a Kruskal–Wallis H test indicated a statistically significant difference in Autonomy Satisfaction between the four finance coach type conditions, *χ*^2^(3) = 19.098, *p* < 0.000, with a mean rank Autonomy Satisfaction score of 2.67. Pairwise comparisons revealed evidence for a difference in the Autonomy Satisfaction scores between the female and the male high-agency finance coach conditions (*p* = 0.038, Bonferroni-adjusted), with the female high-agency finance coach receiving a significantly lower mean Autonomy Satisfaction score (*M* = 2.17, *SD* = 0.88) than the male high-agency finance coach (*M* = 2.72, *SD* = 0.77). There was also strong evidence for a difference between the high-agency female finance coach and the low-agency male finance coach (*p* = 0.005, adjusted using the Bonferroni correction), with the high-agency female finance coach receiving a significantly lower mean Autonomy Satisfaction score (*M* = 2.17, *SD* = 0.88) than the low-agency male finance coach (*M* = 2.95, *SD* = 1.05). Further, there was strong evidence for a difference between the high-agency and the low-agency female finance coach conditions (*p* < 0.001, Bonferroni-adjusted). The high-agency female finance coach resulted in a significantly lower mean Autonomy Satisfaction score (*M* = 2.17, *SD* = 0.88) than the low-agency female finance coach (*M* = 2.96, *SD* = 1.05). There was no evidence of a significant difference in the Autonomy Satisfaction scores between the other conditions.

**Figure 2:**
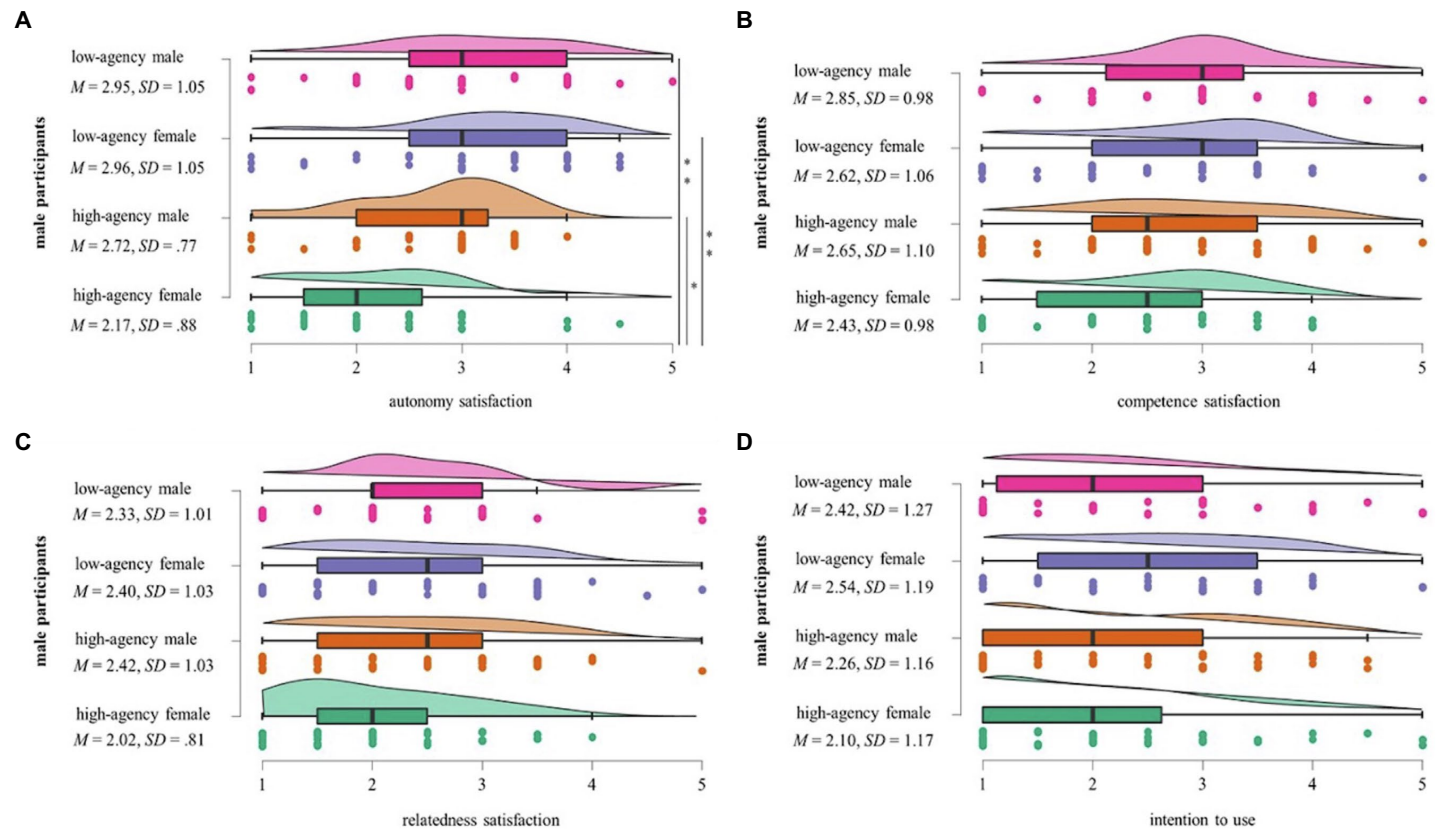
Male participants’ mean scores for the four finance coach type conditions. Significant interactions are demonstrated with a line on the right hand side. One asterisk * demonstrates a significance level of *p* < 0.05 and two asterisks ** demonstrate a significance level of *p* < 0.01.

**Figure 3:**
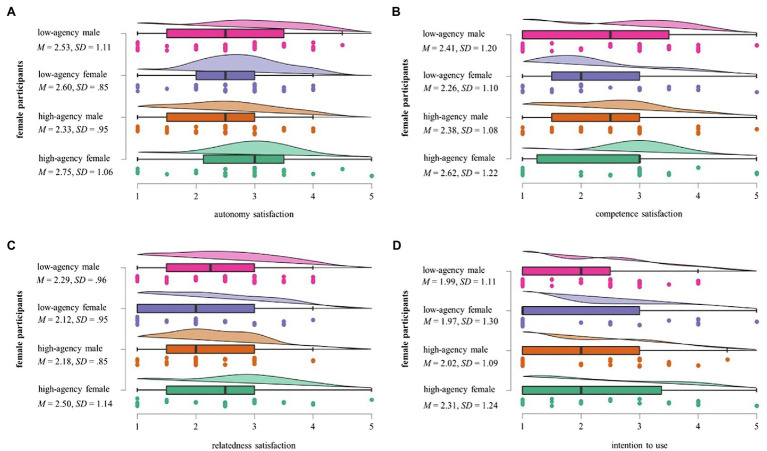
Female participants’ mean scores for the four finance coach type conditions.

For the female participants, there was no statistically significant difference in Autonomy Satisfaction between the four finance coach type conditions, *χ*^2^(3) = 2.639, *p* = 0.451, suggesting that the main effect described in section “Overall Evaluations and Main Effects” is accounted for solely by the varying ratings within the male participant group. Neither within the subgroup of female participants nor in that of male participants did differential assessments of the four finance coach conditions in terms of their Competence Need Satisfaction [*χ*^2^(3) = 2.580, *p* = 0.461 for males; *χ*^2^(3) = 1.181, *p* = 0.758 for females], Relatedness Need Satisfaction [*χ*^2^(3) = 4.185, *p* = 0.242 for males; *χ*^2^(3) = 1.917, *p* = 0.590 for females], or ITU scores [*χ*^2^(3) = 3.447, *p* = 0.328 for males; *χ*^2^(3) = 2.040, *p* = 0.564 for females] reach statistical significance. Descriptive statistics indicate that female participants evaluated the high-agency female AI assistant most favorable across all variables of interest, as reflected by the highest mean scores for satisfaction of all three needs as well as ITU. For male participants, the trends appear to be more mixed: while the low-agency female AI assistant received their highest scores for Competence Satisfaction, Autonomy Satisfaction, and ITU, male users seem to feel most related to the high-agency male AI assistant according to the descriptive statistics. An overview of all descriptive mean values for the ratings of male participants is found in Figure 2, for those of female participants in Figure 3.

### The Mediating Role of Autonomy Need Satisfaction

**Figure 4:**
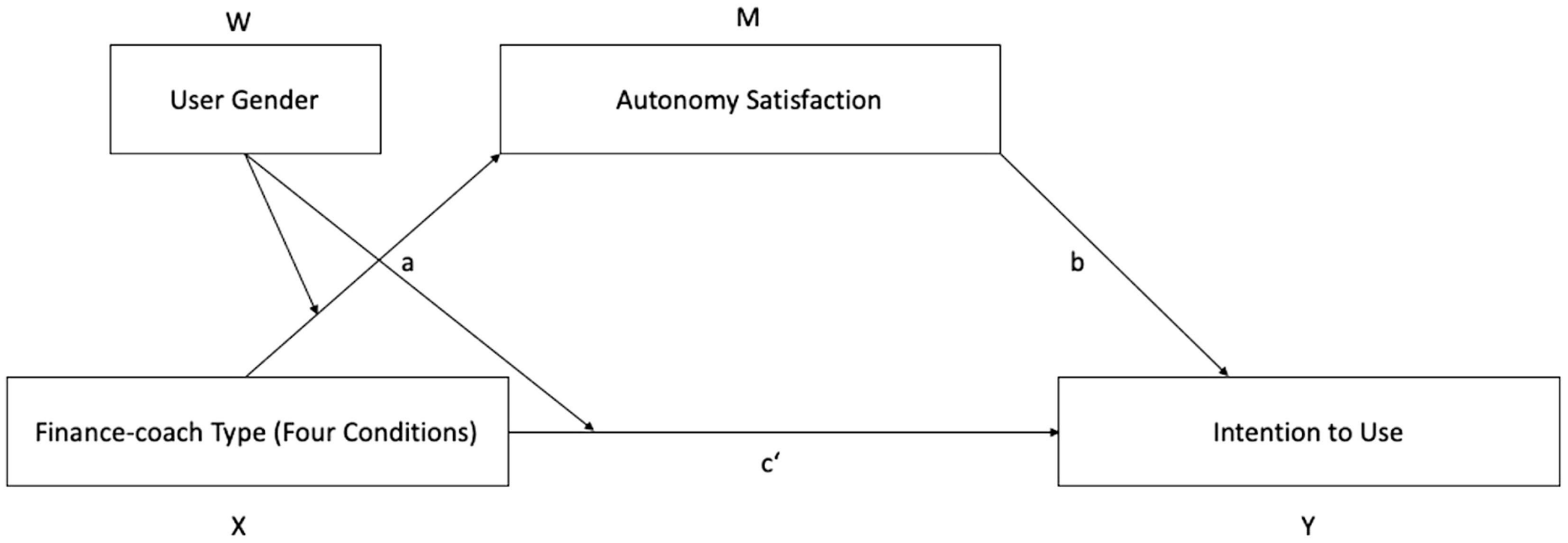
Simple moderated mediation model (Model 8 in the Process macro by Hayes, 2018).

For further analyses, a moderated mediation model was employed using the SPSS PROCESS macro model number 8 (Figure 4; Hayes, 2018). This model tested by participant gender (female vs. male) whether Autonomy Satisfaction mediated the interaction effect of the four finance coach conditions on the ITU (see Table 2). For this analysis, we had to exclude two non-binary participants from the sample in order to fully explore the effect of the moderator (participant gender). For the multi-categorical X variable (finance coach type), indicator coding was used, with X1 comparing the female and the male high-agency finance coaches, X2 comparing the high- and low-agency female finance coaches, and X3 comparing the high-agency female and the low-agency male finance coaches.

**Table 2:** Moderated mediation table for the effect of the finance coach type through autonomy satisfaction, moderated by user gender, on Intention to Use.

Indicator coding	Path	Coeff./effect	SE (HC4)	*t*	*p*	LLCI	ULCI
X1	a _interaction_	0.964	0.322	2.991	0.003	0.330	1.599
	a _female_	−0.417	0.267	−1.561	0.120	−0.942	0.109
	a _male_	0.548	0.181	3.029	0.003	0.192	0.903
	c’ _interaction_	−0.318	0.312	−1.017	0.310	−0.933	0.297
	c’ _female_	0.038	0.235	0.160	0.873	−0.426	0.501
	c’ _male_	−0.280	0.203	−1.377	0.170	−0.680	0.120
X2	a _interaction_	0.940	0.337	2.790	0.006	0.277	1.602
	a _female_	−0.147	0.263	−0.558	0.577	−0.663	0.370
	a _male_	0.793	0.211	3.761	0.000	0.378	1.208
	c’ _interaction_	0.032	0.314	0.101	0.920	−0.587	0.650
	c’ _female_	−0.226	0.249	−0.907	0.365	−0.717	0.265
	c’ _male_	−0.194	0.190	−1.023	0.307	−0.568	0.180
X3	a _interaction_	1.003	0.362	2.769	0.006	0.290	1.717
	a _female_	−0.224	0.277	−0.809	0.419	−0.768	0.321
	a _male_	0.780	0.234	3.330	0.001	0.319	1.240
	c’ _interaction_	−0.160	0.310	−0.517	0.605	−0.769	0.449
	c’ _female_	−0.144	0.212	−0.677	0.499	−0.561	0.274
	c’ _male_	−0.304	0.228	−1.335	0.183	−0.751	0.144
n/a	b	0.793	0.049	16.142	0.000	0.696	0.889

X1, comparison between the high-agency female and the high-agency male finance coach; X2, comparison between the high-agency female and the low-agency female finance coach; X3, comparison between the high-agency female and the low-agency male finance coach.

The index of moderated mediation was significantly different from 0 for all three comparisons: for X1 at 95% CI = [0.248, 1.301] with 5,000 iterations; for X2 at 95% CI = [0.228, 1.263] with 5,000 iterations; and for X3 at 95% CI = [0.225, 1.410] with 5,000 iterations. Significant effects are supported by the absence of zero within the confidence intervals. According to Hayes (2018), a moderated mediation can thus be inferred, indicating that the four AI assistant types affected autonomy satisfaction differently depending on participant gender, and lower autonomy satisfaction in turn led to lower intentions to use the assistant.

The conditional indirect effect of the finance coach type by participant gender *via* Autonomy Satisfaction on ITU was significantly different from 0 for male participants for the female high-agency condition compared to the male high-agency condition: 95% CI = [0.143, 0.727], with 5,000 iterations; for the female high-agency condition vs. the female low-agency condition: 95% CI = [0.291, 0.968], with 5,000 iterations; and for female high-agency condition vs. the male low-agency condition: 95% CI = [0.234, 1.026], with 5,000 iterations. For female participants, the indirect effect of the finance coach type by participant gender *via* Autonomy Satisfaction on ITU was not significantly different from 0.

Within the moderated mediation model, the conditional direct effect of finance coach type on ITU by participant gender was not significant (*p* > 0.05) for all comparisons and both participant genders.

The interaction effect of the *a* path was significant for X1: *p* = 0.003, for X2: *p* = 0.006 and for X3: *p* = 0.006. The interaction explains 4.2% of the variance in Autonomy Satisfaction. The effect of finance coach type on Autonomy Satisfaction according to participant gender showed no significant effect for female participants for all three comparisons (X1-X3). For male participants, the effect was significant for X1 *p* = 0.003; for X2 *p* = 0.000; and for X3 *p* = 0.001.

The effect of Autonomy Satisfaction on ITU was significant (*p* < 0.000). The interaction explains 0.4% of the variance of ITU. The interaction effect for the *c’* path is not significant *p* > 0.05. Thus, the direct effect is not moderated. Overall, the bootstrap results were also robust. Since the model indicates a significant *a* and a significant *b* path but no moderated direct effect, a significant indirect effect can be inferred from it. The relationship between the four finance coach conditions and ITU is fully transmitted *via* Autonomy Satisfaction.

## Discussion

Due to the increasing application of AI speech assistants across different areas of our daily life, important psychological needs should be in the focus of the design of such systems (cf. André et al., 2018). Nonetheless, the potential role of the BPN has been largely neglected in AI research and development. With the current study, we aimed to complement the limited literature on the role of BPN for technology acceptance (Nguyen and Sidorova, 2018; De Vreede et al., 2021; Jiménez-Barreto et al., 2021). In particular, we wanted to contribute to a better understanding of how two specific design parameters of AI assistants are related to the fulfillment of BPNs and how this, in turn, affects behavioral intentions to use a system. By creating four introductory product videos of an AI-based finance coach, we manipulated (a) gender cues (female-sounding vs. male-sounding synthetic voice) and (b) agency (high vs. low), i.e., the system’s degree of control and independent decision-making without the user’s input. Since the female-featured AI assistant with high agency represents characteristics that are stereotypically associated with men (e.g., assertiveness or independent decision-making, cf. Abele et al., 2008), but which should actually be attributable to all genders as a matter of equality, this specific version could also be regarded as a more “feminist” design of AI technology. AI assistants of this type are currently underrepresented on the market (West et al., 2019). However, since both manipulated design factors are frequently discussed in the public discourse on AI (André et al., 2018; West et al., 2019; Laitinen and Sahlgren, 2021) and could be adjusted in many real-world applications, we believe our experiment to be relevant not only for academia but also for practice.

### Discussion of Main Findings

In line with our assumptions, we found strong positive correlations between the extent to which participants perceived their Needs for Autonomy, Competence, and Relatedness to be met and their Intentions to Use the AI assistant. This highlights the relevance of all three BPN for technology acceptance. Overall, men indicated a slightly greater willingness to use the AI-based finance coach across all conditions. Such gender differences are repeatedly reported in the empirical technology acceptance literature (e.g., Powell, 2013; Hulse et al., 2018; McDermott et al., 2020). It has been suggested that lower overall acceptance scores from women may be driven by variables that share variance with gender, such as lower levels of computer-related self-efficacy or higher levels of computer anxiety among female users (cf. Mara and Meyer, 2022). In the context of the present study, additional confounding factors could be related to the fact that women underestimate their financial knowledge (Cannivet, 2018), or to the dominance of men in the finance sector (Burke et al., 2006; Brady et al., 2011).

#### Autonomy Satisfaction (H1)

Accounting for human autonomy in the development and application of Artificial Intelligence is considered a necessity for public AI acceptance (e.g., HLEG-AI, 2018). The assumed relationship between design features of an AI assistant, Autonomy Need Satisfaction, and acceptance was also evident in our study. Overall, group comparisons supported H1 that a high-agency AI assistant leads to lower satisfaction of the Need for Autonomy on the side of the users. These results are consistent with previous research in which autonomously behaving chatbots were perceived as controlling or threatening (Maedche et al., 2019; Stein et al., 2019; Chaves and Gerosa, 2020). A significant moderated mediation model further demonstrated that men were more likely than women to see their Need for Autonomy undermined by the high-agency female finance coach (in comparison to the other versions) and that this perceived lack of Autonomy Satisfaction led to a lower Intention to Use.

Unlike female participants, men in our sample evaluated the two female finance coaches significantly differently, with their Autonomy Need being most satisfied by the low-agency female assistant. Consistent with previous studies (e.g., Guo et al., 2020), this shows a preference of male users for bots with female connotation. A reason why female features were precisely preferred in combination with a passive interaction style could be due to the fact that here, in conformity with traditional stereotypical gender roles (which still play a greater role for men on average, cf., Gustafsson Sendén et al., 2019; Hentschel et al., 2019), the male part is left to make important decisions himself, similar to the traditional image of the female secretary who assists a male manager (West et al., 2019; Weisman and Janardhanan, 2020).

Results from the female sample did not show any significant differences, suggesting that manipulations of the AI assistant have a smaller impact on female than on male Autonomy Satisfaction. Taking a look at the descriptive trends, however, it is interesting to note that women reported the highest mean score in Autonomy Satisfaction precisely in the condition rated lowest by men, namely the female high-agency coach, while they ascribed the least satisfaction to the male high-agency coach. This could indicate that women may feel their Autonomy Need more satisfied by AI assistants of their same gender and perhaps especially by such that correspond to a more contemporary image of women, with which feminism-oriented users could identify (cf., Sternadori and Abitbol, 2019). In section Gender Differences in the Evaluation of (Non-)Stereotypical AI Assistants, we discuss possible explanations for observed gender differences in the evaluation of the AI assistants in more detail. At the same time, we would like to emphasize that further studies are needed, especially to corroborate the tendencies observed for female participants.

#### Relatedness Satisfaction (H2)

In contrast with what we assumed in H2, there were no significant main effects of finance coach type on how much the Need for Relatedness was satisfied. In line with the Similarity Attraction Theory (Byrne, 1971) and with corresponding findings from the field of human-robot interaction (Eyssel et al., 2012), we had predicted that participants would feel more connected and thus experience greater satisfaction of their Need for Relatedness when the AI assistant matched their own gender. While group differences failed to reach a level of statistical significance, the descriptive mean values indicate the expected trend, as women gave the highest Relatedness Satisfaction scores to a female finance coach and men gave the highest scores to a male finance coach. Explanations for these tendencies may also be found in the developmental and social psychological literature on peer relations, which shows that children and adults are still more likely to form connections and spend time with same-gender peers than with other-gender peers, although it is assumed that more balanced orientations toward different genders could help expectations about gender roles become less rigid (Rubin et al., 2016; Bukowski and DeLay, 2020). Furthermore, some empirical work on parasocial relationships with media figures, for example with characters from science fiction movies (Hall, 2019), also suggests a positive influence of gender identification with screen characters on the perceived social connection with them. Parasocial relationships refer to the emotional attachment that people develop towards a media personality or fictional character (Horton and Richard Wohl, 1956) and have recently become a subject of research also in the context of human interactions with AI characters (Noor et al., 2021). We encourage further research with larger sample sizes to examine under which conditions Relatedness is a function of gender-matched human-machine interactions, including in more social application domains of AI than banking.

#### Competence Satisfaction (H3 and H4)

In support of H3, we found a positive correlation between the level of perceived Competence Satisfaction and ITU, highlighting the relevance of BPN for user acceptance. H4 assumed differences in Competence Satisfaction as a function of the AI assistant’s agency, while we did not predict whether participants would regard an agentic AI assistant as either a positive or negative influence on their experience of self-competence (Williams and Lillibridge, 1992). The found positive correlation between the perceived competence of the AI assistant and the fulfillment of the Need for Competence (as well as of the other needs) suggests that overall, our participants did not feel threatened by a competent finance coach, but rather perceived it as a supportive resource. This is in line with a recent study on the introduction of AI systems in the workplace, in which positive associations between a system’s usefulness, perceptions of the system as resource, and the fulfillment of psychological needs were found (Stamate et al., 2021). Generally, in the absence of significant main effects and hence in contradiction to H4, our data indicate that neither the degree to which an AI assistant was perceived as competent nor the Competence Need Satisfaction score on the side of the participants were determined by any of our experimental manipulations. However, taking a closer look at the descriptive statistics, we see similar trends as before. Women, in contrast with men, attributed the highest mean value of perceived competence to the female high-agency finance coach and also considered it to best fulfill their own Need for Competence, whereas men reported the lowest Competence Need Satisfaction for this version and the highest for the low-agency male finance coach. According to literature on gender stereotypes (Bakan, 1966; Abele et al., 2008; Hentschel et al., 2019), the agentic and competent traits are closely related to one another and have traditionally been more often attributed to men than to women, both by male and female raters (cf. Nass et al., 1997). The trends in our study results do not support this, but rather imply that women in our sample attributed more competence and more competence satisfaction potential to the female high-agency finance coach than to the male versions. However, these trends would need to be systematically investigated in follow-up studies with greater statistical power.

#### Gender Differences in the Evaluation of (Non-)Stereotypical AI Assistants

The finding that both male and female users evaluate an AI assistant with a female voice most favorable in our experiment could be explained by a general inclination of people to prefer female voices (Wiley and Eskilson, 1985; Ko et al., 2006). A number of previous studies suggest that participants of different genders perceive female voices more positively and, for example, attribute more likeability or kindness to them as compared to male voices (Ko et al., 2006; Krahé et al., 2021). However, the outcome that men prefer low agency in connection with the female voice, while women show a tendency to prefer high agency, requires closer examination. We propose to view each of the preferred AI assistants as either conforming or non-conforming to stereotypical gender roles. While the low-agency female finance coach, to whom male participants attributed the greatest need satisfaction on average, aligns with the traditional view of women as non-agentic (but communal), the high-agency female finance coach could be regarded as opposing this stereotype and thus also as a more “feminist” concept of a voice assistant. Following this notion, our data would suggest that different gender groups also differ in how they evaluate technology designs that either correspond or do not correspond to gender stereotypes. From this point of view, female participants in our study exhibited a tendency to be more open to the counter-stereotypical combination of highly agentic and female features, while men expressed a clear preference for the stereotype-conforming combination of low agency and female features.

These interpretations are in line with previous research. As the Role Congruity Theory (Eagly and Karau, 2002) suggests, a perceived incongruity between the stereotypical female gender role and agentic leadership roles can lead to a less favorable view of women. In order to be accepted as leaders, women might thus temper their agency with communal characteristics (Schock et al., 2019). Members of all gender groups have been shown to be susceptible to holding negative biases against role-incongruent individuals. In line with our own findings, several studies indicate that males tend to view agentic women more unfavorably than females do. For instance, if women violate traditional norms by presenting themselves as agentic in job resumés, men were found to perceive this particularly negatively (Tyler and McCullough, 2009). Men judged a fictional female politician less likable when the character used agentic (vs. communal) language, whereas there were no systematic differences in the judgments of female participants (Bray et al., 2020). In a survey with United States college students, only 29.6% of male respondents, in comparison with 40.6% of female respondents, somewhat or fully agreed with the statement “Women who are not feminine are good role models” (Duncan et al., 2019). In a study on social learning, even 5-8-year-old boys were shown to be relatively reluctant to accept facts by girls who were introduced as counter-stereotypical experts (e.g., knowing well about construction or football), whereas gender conformity did not matter that much for girls (Boseovski et al., 2016). This is consistent with findings that already at an early age, boys seem to respond less positively to deviations from stereotypical gender norms than girls (Blakemore, 2003). Linking this research to the current results, the existing empirical evidence is in support of the findings from the current study, suggesting that males, on average, are more prone to hold a role-congruent and gender-stereotypical view of women (and perhaps also of themselves). This may provide further explanation as to why male participants in the current study preferred the low-agency female finance coach and had the lowest rating for the non-conforming female high-agency finance coach.

In light of these results, and given the widespread human tendency to perceive machines as social agents (Reeves and Nass, 1996; Nass and Moon, 2000), it seems plausible that reluctance to counter-stereotypical characters in interpersonal relationships may be transferred to human-machine relationships as well. Thus, males, relative to females, might encounter non-conforming female AI assistants with more skepticism and feel more threatened than supported in their BPN. Taking responsibility for independent decisions is related to stereotypically male gender roles. Such gender stereotypes do not have to be static, but are influenced by actual and perceived changes in what roles different genders occupy in society (Eagly et al., 2000). With increasing efforts toward gender equality, a.o. supported by feminist initiatives, it was found that the stereotypical view of women increased in agentic traits in recent years, whereas the male stereotype showed less change in either agentic or communal traits (Gustafsson Sendén et al., 2019; Hentschel et al., 2019). Thus, the finding that male participants evaluated the low-agency female assistant most positively and the high-agency female assistant most negatively on average could also be due to the fact that men are still influenced—even more strongly than women—by stereotypical expectations related to their own gender category and thus cannot easily hand over the role of the active, decisive part to a (female-featured) interaction partner.

Consistent with previous research, women in our experiment did not appear to have as clear preferences as men (Tyler and McCullough, 2009; Boseovski et al., 2016; Bray et al., 2020). In the female subsample, the manipulations of the AI assistant did not lead to any statistically significant differences in judgments. However, there was a tendency for women to rate the counter-stereotypical high-agency assistant with female voice as most favorably across all outcome variables. Although this trend could still be a coincidental finding and thus must be carefully re-assessed by follow-up studies, it should be interesting to reflect on potential underlying motives of female participants. First, their seemingly greater openness to the use of high-agency finance coaches could be associated with individual differences in the perception of the AI assistant as either a competing authority that limits one’s own experience of autonomy, or as a supportive tool for the achievement of shared objectives (cf. Stamate et al., 2021). Against the backdrop of women’s still lower self-efficacy in financial matters (Amatucci and Crawley, 2011; Farrell et al., 2016), women in particular might assume an AI-based finance coach will support them in becoming more autonomous and independent of advice from third parties, rather than narrowing their space for decision-making. Further research could look at potential interaction effects between gender, application context, and perceptions of AI as a helpful resource in relation to BPN satisfaction. Second, women may be more attentive to stereotypical portrayals of their own gender. They might perceive such portrayals as outdated and rate them as less favorably accordingly. Such behavior would likely be associated with positive attitudes toward feminism and a stronger rejection of traditional gender norms. Including such constructs was beyond the scope of this paper, but should be considered in future work. Third, females might prefer the high-agency female assistant, because it is the one, they would most readily identify with. In past studies, women were found to have a stronger tendency to anthropomorphize machines (e.g., Abel et al., 2020) and to identify with fictional screen characters than men (Aytulun and Sunai, 2020). Women who view themselves as independent and assertive might therefore prefer to identify with an artificially intelligent counterpart that corresponds to a more contemporary image of women. This approach is supported by the observation that women in the high-agency female condition of our study also reported the highest Relatedness Need Satisfaction, although again these results merely represent descriptive trends.

### Limitations and Outlook

Besides the contributions of the current study, we also need to note some limitations. First, our experimental manipulations of the AI finance coach had no significant impact on how much participants perceived their Needs for Relatedness and Competence to be met. Moreover, the main effect that we found on Autonomy Satisfaction accounted for differences among the male user group only. Further research is therefore needed to rigorously examine the interesting yet non-significant trends—particularly among female users—that could be observed in our descriptive results. Our study was a priori designed to detect medium to larger effects of the finance-coach video conditions on participant evaluations only. Conceptually similar experiments with greater statistical power and/or stronger manipulations of an AI assistant might therefore be able to reveal further significant effects which we were unable to detect. Tendencies in the mean scores from male versus female participants for both Competence and Relatedness Satisfaction give an indication of this possibility (e.g., among the four conditions, the high-agency female assistant received the lowest descriptive scores for Competence and Relatedness Satisfaction from male participants, but the highest descriptive scores for Competence and Relatedness Satisfaction from female participants).

Second, it turned out that two of our measurement instruments had deficits in their psychometric properties. Due to the poor fit indices of the scale used to examine the perceived dominance of the AI assistant, we were unable to adequately test our hypotheses 5 and 6. Follow-up studies with a valid and reliable instrument could look into potential differences in how dominant users perceive male versus female synthetic voices and high-agency versus low-agency AI assistants. Unfortunately, also the Need Frustration items of the BPNSFS scale (Chen et al., 2015; Heissel et al., 2019) lacked reliability in our study. This could be due to our adaptation of the items to better fit the context of the study and raises the question of whether the scale is applicable in its current form to a technology context. Ideally, a context-specific BPN scale should be created to measure Need Satisfaction with technology. Consistent with this is the limitation that the results of this study were not significant for the Relatedness and Competence items. A scale adapted to technology contexts might result in significant differences with regard to the Competence and Relatedness Needs. Therefore, follow-up research with an adapted scale is encouraged.

Third, it could be argued that the transferability of our results to real-world applications might be limited because we used video vignettes in an online study rather than directly exposing participants to the AI finance coach. Ideally, a laboratory experiment or a more interactive research design should follow the current work to ensure that participants can experience direct interaction with the system and better imagine what a daily use of the finance coach would entail. Similar points could be raised about the synthetic voices that we chose for the experiment. With our strategy of using an existing female-sounding voice based on “Vicki” from AWS Polly (see ttsmp3.com) as a baseline and creating a male-sounding equivalent from it by means of professional audio editing software, we aimed for the highest possible internal validity of our data. Following this approach, we were able to ensure that the voices only differed in their gender cues, while characteristics such as pronunciation quality, speech rate, or affective expression could be held constant. However, this approach also implied that the voice of the male finance coach was unknown to the participants, whereas the female voice was available on the market and might have been implemented in actual applications. Therefore, we cannot rule out that some people may have been familiar with it before taking part in our study. Right now, there are not enough female and male German voices available that are similar enough in the above-mentioned characteristics to be used for experimental research. Even when referring to English text-to-speech systems, female voices are much more commonly used on existing devices and thus have a higher probability of being known than male-sounding voices. The role of familiarity with an AI assistant's voice should therefore be considered in future studies (e.g., as a control variable). Options to achieve comparable levels of familiarity could also include the creation of two new voices or the use of voices that are less commonly used on existing devices.

Fourth, we want to acknowledge that we did apply gender as a categorical variable which leads to several shortcomings, as it does not account for the observation that traditional gender categories have greater variance within than between them. Instead, gender could be conceptualized dimensionally in future studies to be more inclusive. This way, participants do not have to decide between fixed gender categories but have more options in describing their gender identity, leading to more precise measurements (exhaustivity; see Döring, 2013).

Lastly, although this study had a tight focus on BPN Satisfaction and ITU of a speech assistant (AI application) in the banking context, our findings may also apply to other contexts and domains in which AI applications play a role, such as the healthcare sector, online shopping, and various areas related to customer service. Future research should investigate the importance of the BPN in these domains and compare participants’ preferences and levels of Needs Satisfaction when using such AI systems. Further, it would be interesting to examine whether, and to what degree, the differences between male and female participants, as revealed in this study, are also to be found in other domains.

Attempts to incorporate feminist approaches in AI-based applications are still rare today. If gender-stereotypical design preferences are met in everyday devices and traditional images of passive female assistants are re-emerging, further disadvantages for women could be a result. This is particularly relevant in light of the aforementioned research on existing voice assistants, which are frequently criticized for the reproduction of traditional gender stereotypes through the combination of female gender markers with low levels of agency (Broverman et al., 1972). Consequently, the current results are important as they indicate that male participants indeed show a preference for the stereotypical voice assistant, while female participants preferred a counter-stereotypical one. Further producing voice assistants that conform with gender stereotypes could increase outdated perceptions of females as passive servants (West et al., 2019). Therefore, in order to progress toward societal goals of gender equality and fair opportunities, it would be detrimental if manufacturers of such systems, in the interest of potential economic gains, were to make the deduction that male users should be offered low-agency female assistants. In contrast, it would be welcome if the use of AI assistants that are either counter-stereotypical, genderless or not at all resembling humans, could be considered more often for real-world applications.

## Conclusion

A key finding of this study is that the design of an AI assistant for banking influences male users’ Need for Autonomy, which when satisfied, has a positive impact on their behavioral intention to use the AI assistant. Male participants exhibited a preference (highest Autonomy Satisfaction and highest ITU) for the low-agency female AI assistant, while they evaluated the high-agency female version least favorably. Female participants, on the other hand showed no significant differences in their ratings, although descriptive trends point to opposite preferences compared to males.

The different findings for the male and female sub-samples seem to suggest that, in order to maximize customer satisfaction, AI systems should allow users to choose their preferred design characteristics. However, such a simplistic conclusion would not only ignore causes for the observed preferences, but also disregard potential societal implications of a perpetuation of gender stereotypes through technology.

Our study indicates that particularly male participants responded positively to an AI assistant with a female voice that conformed to stereotypical feminine traits. Considering that users have been found to employ degrading language toward female-connotated speech assistants in the past, the propagation of AI technology that meets the outdated stereotype of subservient women would risk further exacerbating such problematic phenomena.

## Data Availability Statement

The original contributions presented in the study are included in the article/Supplementary Material, further inquiries can be directed to the corresponding author.

## Ethics Statement

Ethical review and approval was not required for the study on human participants in accordance with the local legislation and institutional requirements. The patients/participants provided their written informed consent to participate in this study.

## Author Contributions

MM, LM, and SS conceptualized the study. SS and LM created the stimulus materials and performed data collection. LM and MM performed statistical analyses and discussed the results. LM wrote the first draft of the manuscript. All authors contributed to the article and approved the submitted version.

## Funding

The publication of this work was supported by the Open Access Publishing Fund of the Johannes Kepler University Linz.

## Conflict of Interest

The authors declare that the research was conducted in the absence of any commercial or financial relationships that could be construed as a potential conflict of interest.

## Publisher’s Note

All claims expressed in this article are solely those of the authors and do not necessarily represent those of their affiliated organizations, or those of the publisher, the editors and the reviewers. Any product that may be evaluated in this article, or claim that may be made by its manufacturer, is not guaranteed or endorsed by the publisher.
