# Supplementary material for: Do Men Have No Need for “Feminist” Artificial Intelligence? Agentic and Gendered Voice Assistants in the Light of Basic Psychological Needs
Source: Front Psychol. 2022 Jun 14;13:855091. doi: 10.3389/fpsyg.2022.855091 (PMC9239329; doi:10.3389/fpsyg.2022.855091)

12 APPENDIX

Appendix A

Stimuli Text for the Finance Coach Video Sequences

This appendix contains the full text stimuli which were shown to participants within the video sequences. Version one was used in the low-agency conditions and version two was used in the high-agency conditions.

The following is the full text for the high-agency conditions. Bold text passages were shown in text on the screen, underlined passages were shown in bold on the screen. Italic passages were the manipulations for the condition. The remaining passages were not shown and participants only heard these passages through the voice of the finance coach.

Hallo
Ich bin dein künstlich intelligenter Finanzcoach!
Ich helfe dir, deine Sparziele zu erreichen.
Bankgeschäfte werden mit mir zum Kinderspiel.
Du kannst mich mit jedem bestehenden Bankkonto verwenden, wenn du mich **als App am Handy** installierst, stehe ich Rund um die Uhr zur Verfügung. Öffnungszeiten gibt es bei mir nicht. **Ich verbinde mich *selbstständig* mit deinen Kontodaten.**
Ohne dein Zutun **übernehme ich Einzahlungen, Sparvorhaben und andere Aufgaben für dich.**
Viele Menschen sparen weniger als sie gerne würden mit mir als Finanzcoach hat das ein Ende.
Mittels künstlicher Intelligenz führe ich *vollautomatisiert* Analysen über dein Finanzverhalten durch.
Habe ich dich und deine Finanzsituation kennengelernt, ***definiere ich*** **für dich persönliche Sparziele.**
Du gibst vor, bis wann du einen bestimmten Geldbetrag gespart haben möchtest.
Ich *lege* daraufhin passende Wochenziele für dich *fest.*
Immer, wenn ich merke, dass auf deinem Konto genug Geld vorhanden ist, lege ich etwas zur Seite, damit dein Wochenziel erreicht wird.
Dafür ist kein Zutun deinerseits notwendig!
Sparen geht mit mir schnell und unkompliziert. In nur fünf Minuten kann ich bei deiner Bank oder bei einer Partnerbank ein Sparkonto anlegen.
Dein Vorteil: ***Dank meinen Analysen brauchst du dich um nichts mehr zu kümmern. Die Überweisung und Verwahrung der Sparbeträge übernehme ich für dich automatisch.*** So erreichen wir die Ziele und kurbeln deine finanzielle Fitness an.
Mit mir wird Sparen zum Vergnügen!

English translation:

Hello

I am your artificially intelligent Financecoach!

I help you achieve your savings goals.

With me, banking is a walk in the park.

You can use me with any existing bank account, if you install me **as an app on your phone**, I am available around the clock. There are no opening hours with me. **I *independently* connect myself with your account data.**

Without your intervention, **I take care of deposits, savings and other tasks for you.**

Many people save less than they would like to but with me as a financial coach, this comes to an end.

With the use of artificial intelligence, I perform *fully automated* analyses of your financial behavior.

Once I have gotten to know you and your financial situation, ***I define*** **personal savings goals for you.**

You specify by when you want to have saved a certain amount of money.

I then *set* suitable weekly goals for you.

Whenever I notice that there is enough money in your account, I put something aside so that your weekly goal is reached.

For this, *no involvement* from your side is necessary!

Saving with me is quick and easy. In just five minutes I can open a savings account at your bank or at a partner bank.

Your advantage: ***Thanks to my analyses, you don't have to take care of anything. I will automatically take care of the transfer and safekeeping of the savings amounts for you.*** This is how we achieve the goals and boost your financial fitness.

With me, saving becomes a pleasure!

The following is the full text for the low-agency conditions. Bold text passages were shown in text on the screen, underlined passages were shown in bold on the screen. Italic passages were the manipulations for the condition. The remaining passages were not shown and participants only heard these passages through the voice of the finance coach.

Hallo

Ich bin dein künstlich intelligenter Finanzcoach!

Ich helfe dir, deine Sparziele zu erreichen.

Bankgeschäfte werden mit mir zum Kinderspiel.

Du kannst mich mit jedem bestehenden Bankkonto verwenden, wenn du mich **als App am Handy** installierst, stehe ich Rund um die Uhr zur Verfügung. Öffnungszeiten gibt es bei mir nicht.

Verbindest du mich **mit deinen Kontodaten, kann ich dich bei Einzahlungen, Sparvorhaben und anderen Aufgaben *unterstützen*.**

Viele Menschen sparen weniger als sie gerne würden mit mir als Finanzcoach hat das ein Ende.

Mittels künstlicher Intelligenz führe ich *auf deinen Wunsch hin* Analysen über dein Finanzverhalten durch.

Habe ich dich und deine Finanzsituation kennengelernt, ***empfehle ich*** **dir persönliche Sparziele.**

Du gibst vor, bis wann du einen bestimmten Geldbetrag gespart haben möchtest.
Ich zeige dir **daraufhin passende Wochenziele.**
Immer, wenn ich merke, dass auf deinem Konto genug Geld vorhanden ist, lege ich etwas zur Seite, damit dein Wochenziel erreicht wird.
Dafür ist eine Bestätigung deinerseits notwendig!
Sparen geht mit mir schnell und unkompliziert. In nur fünf Minuten kann ich bei deiner Bank oder bei einer Partnerbank ein Sparkonto anlegen.
Dein Vorteil: ***Anhand meiner Vorschläge wählst du deine Präferenzen. Sobald du diese bestätigst, beginne ich mit der Überweisung und Verwahrung deiner festgelegten Sparbeträge.*** So erreichen wir die Ziele und kurbeln deine finanzielle Fitness an.
Mit mir wird Sparen zum Vergnügen!

English translation:

Hello
I am your artificially intelligent Financecoach!
I help you achieve your savings goals.
With me, banking is a walk in the park.
You can use me with any existing bank account, if you install me **as an app on your phone**, I am available around the clock. There are no opening hours with me.
If you connect me **with your account data, I can** ***support*** **you with deposits, savings plans and other tasks.**
Many people save less than they would like to - with me as a financial coach, this comes to an end.
With the use of artificial intelligence, I can analyze your financial behavior ***at your request.***
Once I have gotten to know you and your financial situation, ***I recommend*** **personal savings goals.**
You specify by when you want to have saved a certain amount of money.
I then show you suitable weekly goals.
Whenever I notice that there is enough money in your account, I put something aside so that your weekly goal is reached.
For this, a confirmation from your side is necessary!
Saving with me is fast and uncomplicated. In just five minutes, I can open a savings account at your bank or at a partner bank.
Your advantage: ***You choose your preferences based on my suggestions. As soon as you confirm them, I will start the transfer and safekeeping of your specified savings amounts.***
This is how we achieve the goals and boost your financial fitness.
With me, saving becomes a pleasure!

Appendix B

Adapted German Basic Psychological Needs Satisfaction and Frustration Scale

This appendix contains the German adapted version of the Basic Psychological Need and Frustration Scale as used in the study.

Stellen Sie sich nun vor, Sie hätten den Finanzcoach als App auf ihrem Handy oder Computer installiert und das Programm würde wie im Video beschrieben arbeiten. Wie würde sich das für Sie anfühlen?

Würde ich den Finanzcoach für meine Bankgeschäfte nutzen, …

... hätte ich das Gefühl, dass ich selber die Entscheidungen treffen kann und diese meinem eigenen Willen entsprechen. (AS)

... würde ich mich kompetent fühlen, meine Bankgeschäfte zu erledigen. (CS)

... hätte ich das Gefühl, dass dem Finanzcoach meine Anliegen wichtig sind. (RS)

... würde ich mich gezwungen fühlen, Dinge zu tun, die ich mir selbst nicht ausgesucht hätte. (AF)

... würde ich an meiner persönlichen Kompetenz zweifeln, weil mir im Vergleich zum Finanzcoach mehr Fehler bei der Ausführung meiner Bankgeschäfte unterlaufen. (CF)

... würde ich mich aus einer Gruppe, zu der ich gehören möchte, ausgeschlossen fühlen (z.B. Menschen, die ihre Finanzen selbst regeln; Bankkunden, die persönlich betreut werden, etc.). (RF)

... hätte ich das Gefühl, dass der Finanzcoach etwas für mich tut, was meinen eigenen Interessen entspricht. (AS)

... würde ich mich kompetent fühlen, meine Ziele erreichen zu können. (CS)

... würde ich mit der Zeit ein freundschaftliches Gefühl für den Finanzcoach empfinden. (RS)

... würden sich die Aktionen, die ich durchführe, so anfühlen, als ob ich diese tun müsste. (AF)

... wäre ich von meinen eigenen Kenntnissen und Leistungen im Bankingbereich enttäuscht. (CF)

... hätte ich das Gefühl, dass der Finanzcoach sich mir gegenüber kalt und distanziert verhält. (RF)

AS = Autonomy Satisfaction Item, AF = Autonomy Frustration Item,
CS = Competence Satisfaction Item, CF = Competence Frustration Item,
RS = Relatedness Satisfaction Item, RF = Relatedness Frustration Item

Appendix C

Raincloud Graphs to Depict the Descriptives for the Main Effects

Figure C1

Mean Autonomy Satisfaction For The Four Finance-coach Type Conditions

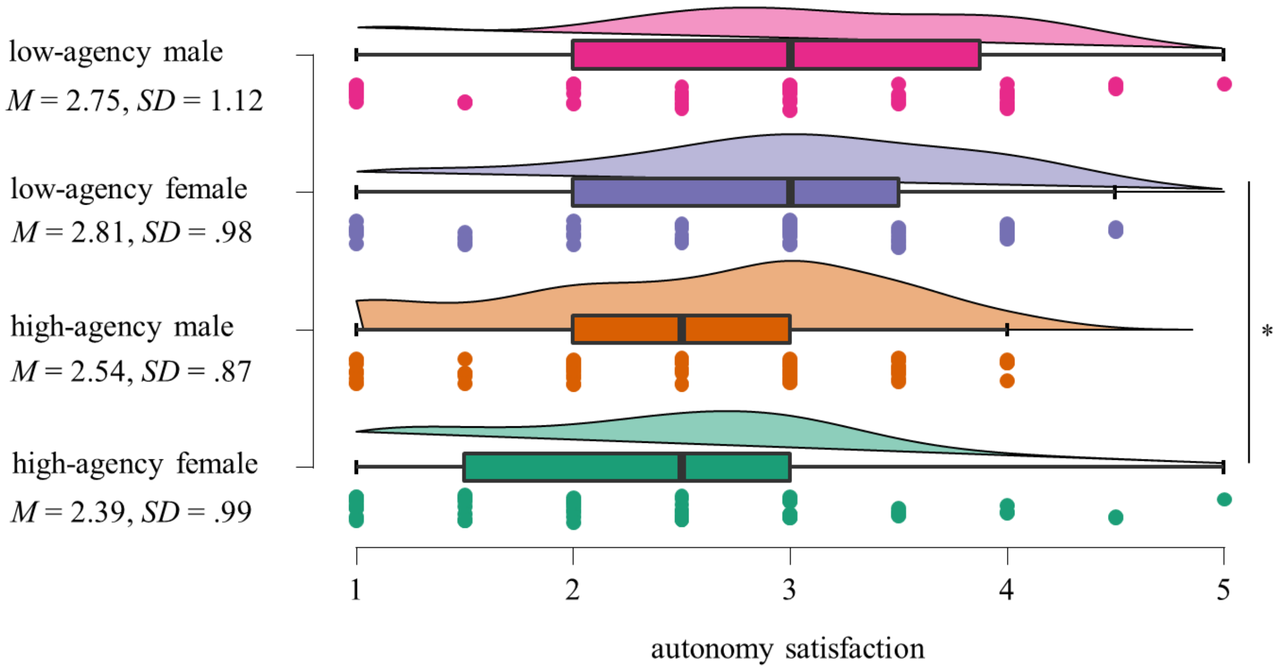

Significant interactions are demonstrated with a line on the right hand side. One star demonstrates a significance level of $p < .05$.

Figure C2

Mean Competence Satisfaction For The Four Finance-coach Type Conditions

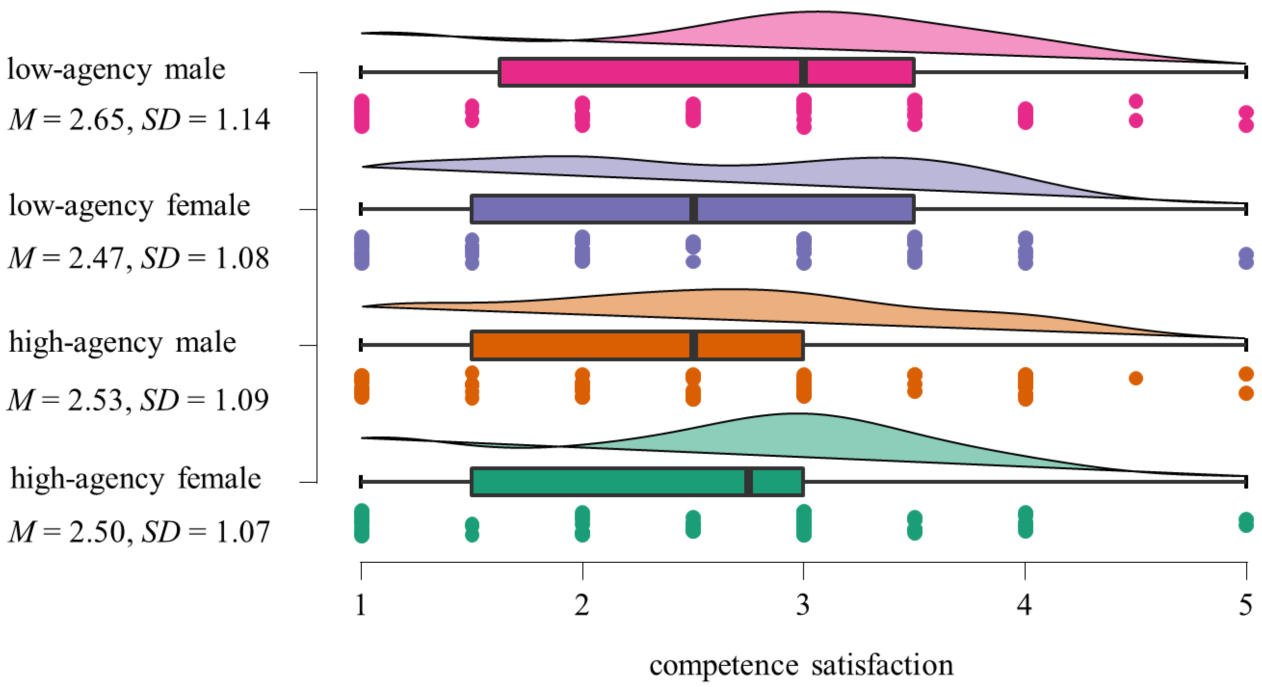

Figure C3

Mean Relatedness Satisfaction For The Four Finance-coach Type Conditions

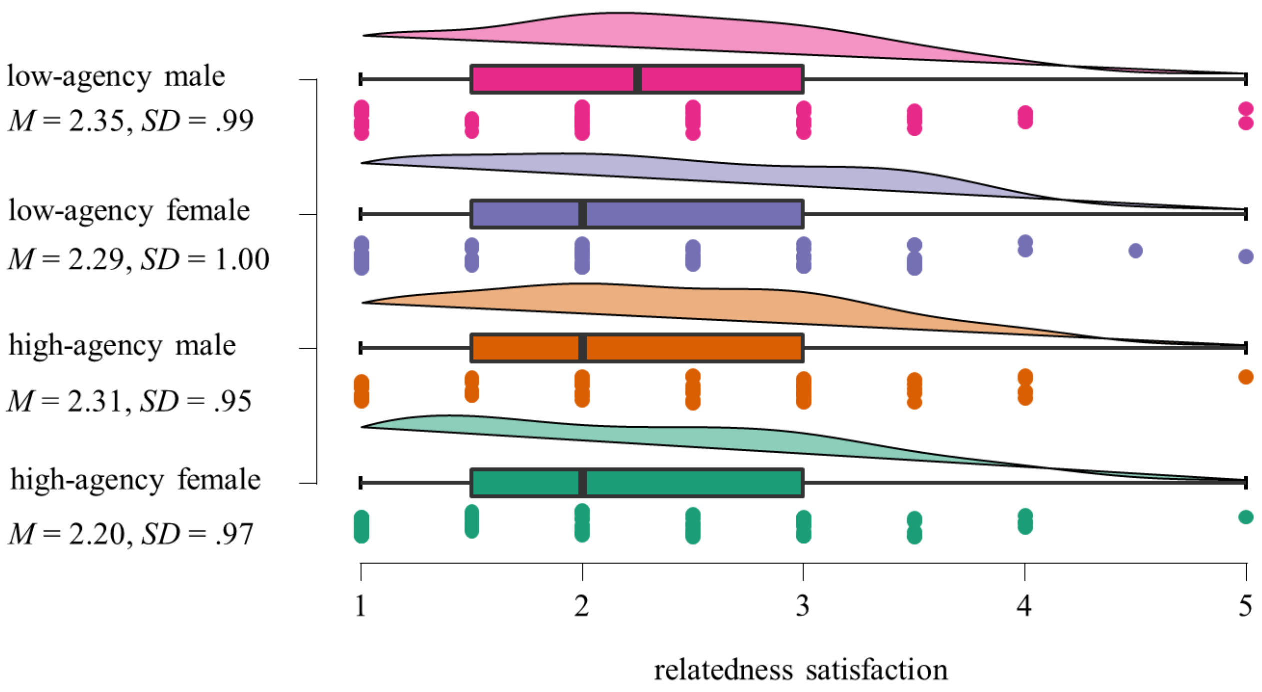

Figure C4

Mean Intention to Use Scores For The Four Finance-coach Type Conditions

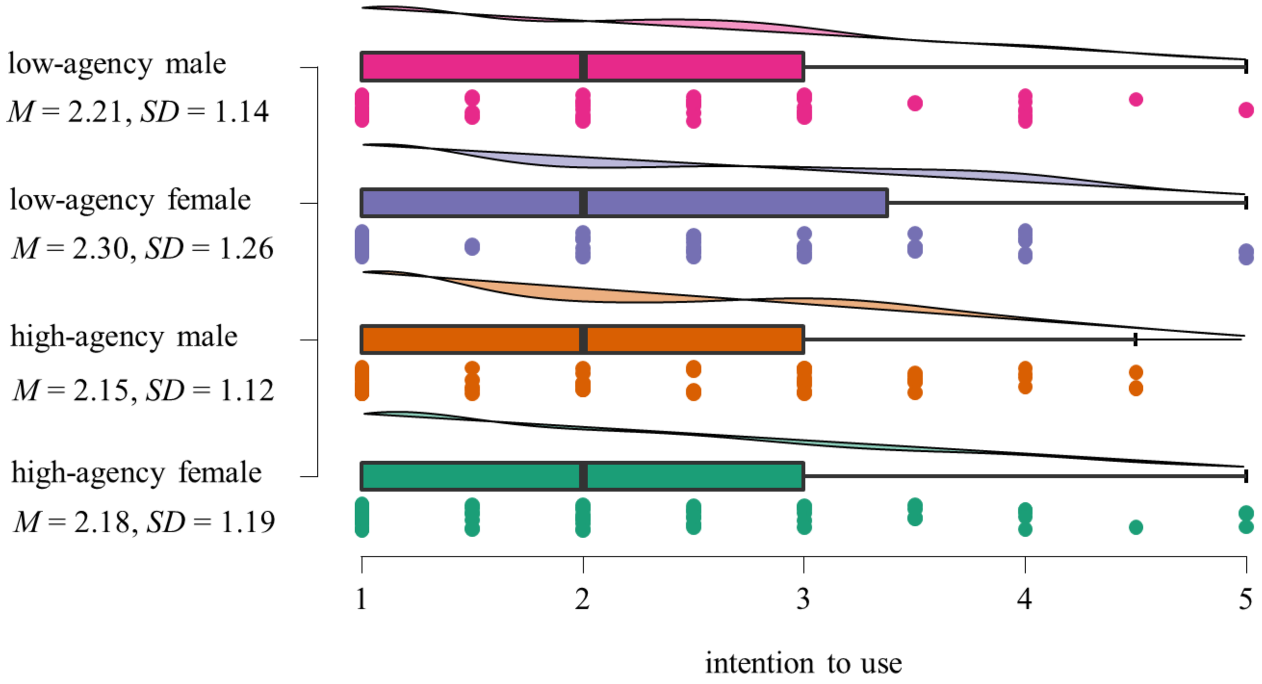

Supplement: Supplementary file 1 [file Data_Sheet_1.pdf]
